# Supplementary figures and images for: Effects of chlorpyrifos on the crustacean Litopenaeus vannamei
Source: PLoS One. 2020 Apr 13;15(4):e0231310. doi: 10.1371/journal.pone.0231310 (PMC7153863; doi:10.1371/journal.pone.0231310)

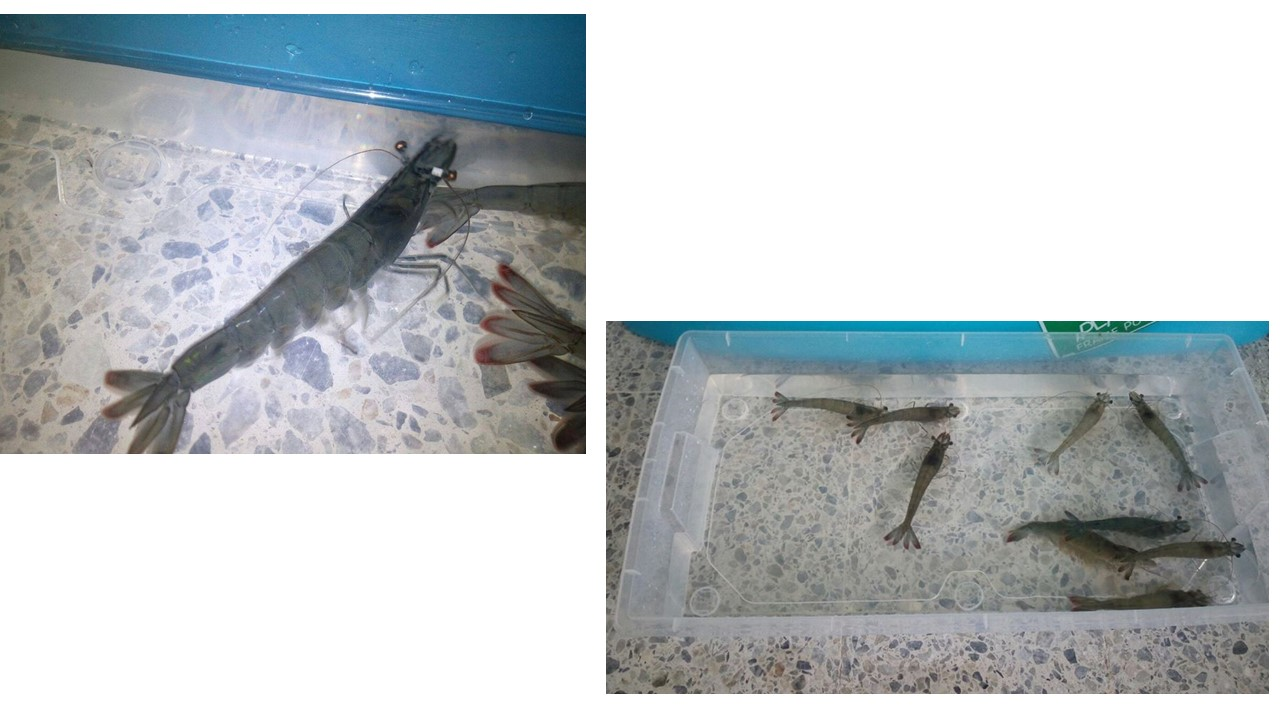

Supplement: S1 Fig — (TIF) [file pone.0231310.s001.tif]
