# Supplementary material for: Effects of chlorpyrifos on the crustacean Litopenaeus vannamei
Source: PLoS One. 2020 Apr 13;15(4):e0231310. doi: 10.1371/journal.pone.0231310 (PMC7153863; doi:10.1371/journal.pone.0231310)
Supplement: S1 File — (DOCX) [file pone.0231310.s002.docx]

ONEWAY Ace CatH CatG CatM GpxH GpxG GpxM GSTH GSTG GSTM LPOH LPOG LPOM BY fact

/STATISTICS DESCRIPTIVES HOMOGENEITY

/MISSING ANALYSIS

/POSTHOC=TUKEY ALPHA(0.05).

**Oneway**

[DataSet0] C:\Users\cortazar\Documents\edisson\edisson5.sav

| **Descriptives** | | | | | | | | | |
| --- | --- | --- | --- | --- | --- | --- | --- | --- | --- |
|  | | N | Mean | Std. Deviation | Std. Error | 95% Confidence Interval for Mean | | Minimum | Maximum |
|  |  |  |  |  |  | Lower Bound | Upper Bound |  |  |
| Ace | 1 | 3 | 26,0000 | 2,32594 | 1,34288 | 20,2220 | 31,7780 | 23,50 | 28,10 |
|  | 2 | 3 | 18,0000 | 2,70555 | 1,56205 | 11,2790 | 24,7210 | 15,20 | 20,60 |
|  | 3 | 3 | 14,0000 | 2,40624 | 1,38924 | 8,0226 | 19,9774 | 11,50 | 16,30 |
|  | Total | 9 | 19,3333 | 5,71227 | 1,90409 | 14,9425 | 23,7242 | 11,50 | 28,10 |
| CatH | 1 | 3 | ,014000 | ,0029052 | ,0016773 | ,006783 | ,021217 | ,0112 | ,0170 |
|  | 2 | 3 | ,019000 | ,0020000 | ,0011547 | ,014032 | ,023968 | ,0170 | ,0210 |
|  | 3 | 3 | ,028000 | ,0070000 | ,0040415 | ,010611 | ,045389 | ,0210 | ,0350 |
|  | Total | 9 | ,020333 | ,0072877 | ,0024292 | ,014732 | ,025935 | ,0112 | ,0350 |
| CatG | 1 | 3 | ,039000 | ,0030000 | ,0017321 | ,031548 | ,046452 | ,0360 | ,0420 |
|  | 2 | 3 | ,041000 | ,0052915 | ,0030551 | ,027855 | ,054145 | ,0370 | ,0470 |
|  | 3 | 3 | ,045000 | ,0043589 | ,0025166 | ,034172 | ,055828 | ,0400 | ,0480 |
|  | Total | 9 | ,041667 | ,0045826 | ,0015275 | ,038144 | ,045189 | ,0360 | ,0480 |
| CatM | 1 | 3 | ,011000 | ,0020000 | ,0011547 | ,006032 | ,015968 | ,0090 | ,0130 |
|  | 2 | 3 | ,012000 | ,0036056 | ,0020817 | ,003043 | ,020957 | ,0090 | ,0160 |
|  | 3 | 3 | ,014000 | ,0030000 | ,0017321 | ,006548 | ,021452 | ,0110 | ,0170 |
|  | Total | 9 | ,012333 | ,0028723 | ,0009574 | ,010126 | ,014541 | ,0090 | ,0170 |
| GpxH | 1 | 3 | 8,4000 | 1,40000 | ,80829 | 4,9222 | 11,8778 | 7,40 | 10,00 |
|  | 2 | 3 | 10,2000 | 2,21133 | 1,27671 | 4,7067 | 15,6933 | 8,50 | 12,70 |
|  | 3 | 3 | 11,7000 | 1,63707 | ,94516 | 7,6333 | 15,7667 | 10,30 | 13,50 |
|  | Total | 9 | 10,1000 | 2,10476 | ,70159 | 8,4821 | 11,7179 | 7,40 | 13,50 |
| GpxG | 1 | 3 | 9,8000 | 1,92873 | 1,11355 | 5,0088 | 14,5912 | 8,40 | 12,00 |
|  | 2 | 3 | 10,4000 | 2,33880 | 1,35031 | 4,5901 | 16,2099 | 9,00 | 13,10 |
|  | 3 | 3 | 11,6000 | 2,51197 | 1,45029 | 5,3599 | 17,8401 | 10,10 | 14,50 |
|  | Total | 9 | 10,6000 | 2,12250 | ,70750 | 8,9685 | 12,2315 | 8,40 | 14,50 |
| GpxM | 1 | 3 | 7,4000 | 1,83576 | 1,05987 | 2,8397 | 11,9603 | 6,10 | 9,50 |
|  | 2 | 3 | 8,1000 | ,95394 | ,55076 | 5,7303 | 10,4697 | 7,50 | 9,20 |
|  | 3 | 3 | 9,3000 | 1,38564 | ,80000 | 5,8579 | 12,7421 | 8,50 | 10,90 |
|  | Total | 9 | 8,2667 | 1,49750 | ,49917 | 7,1156 | 9,4177 | 6,10 | 10,90 |
| GSTH | 1 | 3 | 37,2000 | 5,11175 | 2,95127 | 24,5017 | 49,8983 | 34,10 | 43,10 |
|  | 2 | 3 | 45,6000 | 6,14410 | 3,54730 | 30,3372 | 60,8628 | 41,10 | 52,60 |
|  | 3 | 3 | 52,3000 | 9,26984 | 5,35195 | 29,2724 | 75,3276 | 43,40 | 61,90 |
|  | Total | 9 | 45,0333 | 8,96577 | 2,98859 | 38,1416 | 51,9250 | 34,10 | 61,90 |
| GSTG | 1 | 3 | 126,2000 | 10,18381 | 5,87963 | 100,9020 | 151,4980 | 117,10 | 137,20 |
|  | 2 | 3 | 132,2000 | 9,34077 | 5,39290 | 108,9962 | 155,4038 | 123,70 | 142,20 |
|  | 3 | 3 | 143,2000 | 12,27233 | 7,08543 | 112,7139 | 173,6861 | 134,30 | 157,20 |
|  | Total | 9 | 133,8667 | 11,88034 | 3,96011 | 124,7346 | 142,9987 | 117,10 | 157,20 |
| GSTM | 1 | 3 | 33,4000 | 3,74700 | 2,16333 | 24,0919 | 42,7081 | 30,40 | 37,60 |
|  | 2 | 3 | 41,4000 | 4,39204 | 2,53574 | 30,4896 | 52,3104 | 37,40 | 46,10 |
|  | 3 | 3 | 43,2000 | 6,32139 | 3,64966 | 27,4968 | 58,9032 | 37,20 | 49,80 |
|  | Total | 9 | 39,3333 | 6,22354 | 2,07451 | 34,5495 | 44,1172 | 30,40 | 49,80 |
| LPOH | 1 | 3 | 2,3000 | ,43589 | ,25166 | 1,2172 | 3,3828 | 2,00 | 2,80 |
|  | 2 | 3 | 3,5000 | ,91652 | ,52915 | 1,2233 | 5,7767 | 2,70 | 4,50 |
|  | 3 | 3 | 4,1000 | ,91652 | ,52915 | 1,8233 | 6,3767 | 3,30 | 5,10 |
|  | Total | 9 | 3,3000 | 1,04762 | ,34921 | 2,4947 | 4,1053 | 2,00 | 5,10 |
| LPOG | 1 | 3 | ,083000 | ,0111355 | ,0064291 | ,055338 | ,110662 | ,0730 | ,0950 |
|  | 2 | 3 | ,105000 | ,0232594 | ,0134288 | ,047220 | ,162780 | ,0800 | ,1260 |
|  | 3 | 3 | ,151000 | ,0304138 | ,0175594 | ,075448 | ,226552 | ,1310 | ,1860 |
|  | Total | 9 | ,113000 | ,0360624 | ,0120208 | ,085280 | ,140720 | ,0730 | ,1860 |
| LPOM | 1 | 3 | ,062000 | ,0124900 | ,0072111 | ,030973 | ,093027 | ,0520 | ,0760 |
|  | 2 | 3 | ,094000 | ,0226495 | ,0130767 | ,037736 | ,150264 | ,0730 | ,1180 |
|  | 3 | 3 | ,131000 | ,0192873 | ,0111355 | ,083088 | ,178912 | ,1170 | ,1530 |
|  | Total | 9 | ,095667 | ,0339779 | ,0113260 | ,069549 | ,121784 | ,0520 | ,1530 |

| **Test of Homogeneity of Variances** | | | | |
| --- | --- | --- | --- | --- |
|  | Levene Statistic | df1 | df2 | Sig. |
| Ace | ,024 | 2 | 6 | ,976 |
| CatH | 1,393 | 2 | 6 | ,319 |
| CatG | 1,000 | 2 | 6 | ,422 |
| CatM | ,600 | 2 | 6 | ,579 |
| GpxH | ,597 | 2 | 6 | ,580 |
| GpxG | ,288 | 2 | 6 | ,760 |
| GpxM | 1,339 | 2 | 6 | ,330 |
| GSTH | ,444 | 2 | 6 | ,661 |
| GSTG | ,273 | 2 | 6 | ,770 |
| GSTM | ,361 | 2 | 6 | ,711 |
| LPOH | ,901 | 2 | 6 | ,455 |
| LPOG | 2,041 | 2 | 6 | ,211 |
| LPOM | ,550 | 2 | 6 | ,604 |

| **ANOVA** | | | | | | |
| --- | --- | --- | --- | --- | --- | --- |
|  | | Sum of Squares | df | Mean Square | F | Sig. |
| Ace | Between Groups | 224,000 | 2 | 112,000 | 18,143 | ,003 |
|  | Within Groups | 37,040 | 6 | 6,173 |  |  |
|  | Total | 261,040 | 8 |  |  |  |
| CatH | Between Groups | ,000 | 2 | ,000 | 7,373 | ,024 |
|  | Within Groups | ,000 | 6 | ,000 |  |  |
|  | Total | ,000 | 8 |  |  |  |
| CatG | Between Groups | ,000 | 2 | ,000 | 1,500 | ,296 |
|  | Within Groups | ,000 | 6 | ,000 |  |  |
|  | Total | ,000 | 8 |  |  |  |
| CatM | Between Groups | ,000 | 2 | ,000 | ,808 | ,489 |
|  | Within Groups | ,000 | 6 | ,000 |  |  |
|  | Total | ,000 | 8 |  |  |  |
| GpxH | Between Groups | 16,380 | 2 | 8,190 | 2,578 | ,156 |
|  | Within Groups | 19,060 | 6 | 3,177 |  |  |
|  | Total | 35,440 | 8 |  |  |  |
| GpxG | Between Groups | 5,040 | 2 | 2,520 | ,488 | ,636 |
|  | Within Groups | 31,000 | 6 | 5,167 |  |  |
|  | Total | 36,040 | 8 |  |  |  |
| GpxM | Between Groups | 5,540 | 2 | 2,770 | 1,340 | ,330 |
|  | Within Groups | 12,400 | 6 | 2,067 |  |  |
|  | Total | 17,940 | 8 |  |  |  |
| GSTH | Between Groups | 343,460 | 2 | 171,730 | 3,439 | ,101 |
|  | Within Groups | 299,620 | 6 | 49,937 |  |  |
|  | Total | 643,080 | 8 |  |  |  |
| GSTG | Between Groups | 446,000 | 2 | 223,000 | 1,959 | ,221 |
|  | Within Groups | 683,140 | 6 | 113,857 |  |  |
|  | Total | 1129,140 | 8 |  |  |  |
| GSTM | Between Groups | 163,280 | 2 | 81,640 | 3,342 | ,106 |
|  | Within Groups | 146,580 | 6 | 24,430 |  |  |
|  | Total | 309,860 | 8 |  |  |  |
| LPOH | Between Groups | 5,040 | 2 | 2,520 | 4,043 | ,077 |
|  | Within Groups | 3,740 | 6 | ,623 |  |  |
|  | Total | 8,780 | 8 |  |  |  |
| LPOG | Between Groups | ,007 | 2 | ,004 | 6,815 | ,029 |
|  | Within Groups | ,003 | 6 | ,001 |  |  |
|  | Total | ,010 | 8 |  |  |  |
| LPOM | Between Groups | ,007 | 2 | ,004 | 10,308 | ,011 |
|  | Within Groups | ,002 | 6 | ,000 |  |  |
|  | Total | ,009 | 8 |  |  |  |

**Post Hoc Tests**

| **Multiple Comparisons** | | | | | | | |
| --- | --- | --- | --- | --- | --- | --- | --- |
| Tukey HSD | | | | | | | |
| Dependent Variable | (I) fact | (J) fact | Mean Difference (I-J) | Std. Error | Sig. | 95% Confidence Interval | |
|  |  |  |  |  |  | Lower Bound | Upper Bound |
| Ace | 1 | 2 | 8,00000^*^ | 2,02868 | ,018 | 1,7754 | 14,2246 |
|  |  | 3 | 12,00000^*^ | 2,02868 | ,003 | 5,7754 | 18,2246 |
|  | 2 | 1 | -8,00000^*^ | 2,02868 | ,018 | -14,2246 | -1,7754 |
|  |  | 3 | 4,00000 | 2,02868 | ,200 | -2,2246 | 10,2246 |
|  | 3 | 1 | -12,00000^*^ | 2,02868 | ,003 | -18,2246 | -5,7754 |
|  |  | 2 | -4,00000 | 2,02868 | ,200 | -10,2246 | 2,2246 |
| CatH | 1 | 2 | -,0050000 | ,0036950 | ,420 | -,016337 | ,006337 |
|  |  | 3 | -,0140000^*^ | ,0036950 | ,021 | -,025337 | -,002663 |
|  | 2 | 1 | ,0050000 | ,0036950 | ,420 | -,006337 | ,016337 |
|  |  | 3 | -,0090000 | ,0036950 | ,111 | -,020337 | ,002337 |
|  | 3 | 1 | ,0140000^*^ | ,0036950 | ,021 | ,002663 | ,025337 |
|  |  | 2 | ,0090000 | ,0036950 | ,111 | -,002337 | ,020337 |
| CatG | 1 | 2 | -,0020000 | ,0035277 | ,842 | -,012824 | ,008824 |
|  |  | 3 | -,0060000 | ,0035277 | ,280 | -,016824 | ,004824 |
|  | 2 | 1 | ,0020000 | ,0035277 | ,842 | -,008824 | ,012824 |
|  |  | 3 | -,0040000 | ,0035277 | ,530 | -,014824 | ,006824 |
|  | 3 | 1 | ,0060000 | ,0035277 | ,280 | -,004824 | ,016824 |
|  |  | 2 | ,0040000 | ,0035277 | ,530 | -,006824 | ,014824 |
| CatM | 1 | 2 | -,0010000 | ,0024037 | ,910 | -,008375 | ,006375 |
|  |  | 3 | -,0030000 | ,0024037 | ,471 | -,010375 | ,004375 |
|  | 2 | 1 | ,0010000 | ,0024037 | ,910 | -,006375 | ,008375 |
|  |  | 3 | -,0020000 | ,0024037 | ,699 | -,009375 | ,005375 |
|  | 3 | 1 | ,0030000 | ,0024037 | ,471 | -,004375 | ,010375 |
|  |  | 2 | ,0020000 | ,0024037 | ,699 | -,005375 | ,009375 |
| GpxH | 1 | 2 | -1,80000 | 1,45526 | ,477 | -6,2651 | 2,6651 |
|  |  | 3 | -3,30000 | 1,45526 | ,137 | -7,7651 | 1,1651 |
|  | 2 | 1 | 1,80000 | 1,45526 | ,477 | -2,6651 | 6,2651 |
|  |  | 3 | -1,50000 | 1,45526 | ,586 | -5,9651 | 2,9651 |
|  | 3 | 1 | 3,30000 | 1,45526 | ,137 | -1,1651 | 7,7651 |
|  |  | 2 | 1,50000 | 1,45526 | ,586 | -2,9651 | 5,9651 |
| GpxG | 1 | 2 | -,60000 | 1,85592 | ,945 | -6,2945 | 5,0945 |
|  |  | 3 | -1,80000 | 1,85592 | ,620 | -7,4945 | 3,8945 |
|  | 2 | 1 | ,60000 | 1,85592 | ,945 | -5,0945 | 6,2945 |
|  |  | 3 | -1,20000 | 1,85592 | ,801 | -6,8945 | 4,4945 |
|  | 3 | 1 | 1,80000 | 1,85592 | ,620 | -3,8945 | 7,4945 |
|  |  | 2 | 1,20000 | 1,85592 | ,801 | -4,4945 | 6,8945 |
| GpxM | 1 | 2 | -,70000 | 1,17379 | ,827 | -4,3015 | 2,9015 |
|  |  | 3 | -1,90000 | 1,17379 | ,309 | -5,5015 | 1,7015 |
|  | 2 | 1 | ,70000 | 1,17379 | ,827 | -2,9015 | 4,3015 |
|  |  | 3 | -1,20000 | 1,17379 | ,591 | -4,8015 | 2,4015 |
|  | 3 | 1 | 1,90000 | 1,17379 | ,309 | -1,7015 | 5,5015 |
|  |  | 2 | 1,20000 | 1,17379 | ,591 | -2,4015 | 4,8015 |
| GSTH | 1 | 2 | -8,40000 | 5,76984 | ,374 | -26,1035 | 9,3035 |
|  |  | 3 | -15,10000 | 5,76984 | ,088 | -32,8035 | 2,6035 |
|  | 2 | 1 | 8,40000 | 5,76984 | ,374 | -9,3035 | 26,1035 |
|  |  | 3 | -6,70000 | 5,76984 | ,516 | -24,4035 | 11,0035 |
|  | 3 | 1 | 15,10000 | 5,76984 | ,088 | -2,6035 | 32,8035 |
|  |  | 2 | 6,70000 | 5,76984 | ,516 | -11,0035 | 24,4035 |
| GSTG | 1 | 2 | -6,00000 | 8,71232 | ,778 | -32,7318 | 20,7318 |
|  |  | 3 | -17,00000 | 8,71232 | ,205 | -43,7318 | 9,7318 |
|  | 2 | 1 | 6,00000 | 8,71232 | ,778 | -20,7318 | 32,7318 |
|  |  | 3 | -11,00000 | 8,71232 | ,464 | -37,7318 | 15,7318 |
|  | 3 | 1 | 17,00000 | 8,71232 | ,205 | -9,7318 | 43,7318 |
|  |  | 2 | 11,00000 | 8,71232 | ,464 | -15,7318 | 37,7318 |
| GSTM | 1 | 2 | -8,00000 | 4,03567 | ,197 | -20,3826 | 4,3826 |
|  |  | 3 | -9,80000 | 4,03567 | ,112 | -22,1826 | 2,5826 |
|  | 2 | 1 | 8,00000 | 4,03567 | ,197 | -4,3826 | 20,3826 |
|  |  | 3 | -1,80000 | 4,03567 | ,898 | -14,1826 | 10,5826 |
|  | 3 | 1 | 9,80000 | 4,03567 | ,112 | -2,5826 | 22,1826 |
|  |  | 2 | 1,80000 | 4,03567 | ,898 | -10,5826 | 14,1826 |
| LPOH | 1 | 2 | -1,20000 | ,64464 | ,230 | -3,1779 | ,7779 |
|  |  | 3 | -1,80000 | ,64464 | ,070 | -3,7779 | ,1779 |
|  | 2 | 1 | 1,20000 | ,64464 | ,230 | -,7779 | 3,1779 |
|  |  | 3 | -,60000 | ,64464 | ,643 | -2,5779 | 1,3779 |
|  | 3 | 1 | 1,80000 | ,64464 | ,070 | -,1779 | 3,7779 |
|  |  | 2 | ,60000 | ,64464 | ,643 | -1,3779 | 2,5779 |
| LPOG | 1 | 2 | -,0220000 | ,0187972 | ,511 | -,079675 | ,035675 |
|  |  | 3 | -,0680000^*^ | ,0187972 | ,026 | -,125675 | -,010325 |
|  | 2 | 1 | ,0220000 | ,0187972 | ,511 | -,035675 | ,079675 |
|  |  | 3 | -,0460000 | ,0187972 | ,109 | -,103675 | ,011675 |
|  | 3 | 1 | ,0680000^*^ | ,0187972 | ,026 | ,010325 | ,125675 |
|  |  | 2 | ,0460000 | ,0187972 | ,109 | -,011675 | ,103675 |
| LPOM | 1 | 2 | -,0320000 | ,0152096 | ,169 | -,078667 | ,014667 |
|  |  | 3 | -,0690000^*^ | ,0152096 | ,009 | -,115667 | -,022333 |
|  | 2 | 1 | ,0320000 | ,0152096 | ,169 | -,014667 | ,078667 |
|  |  | 3 | -,0370000 | ,0152096 | ,111 | -,083667 | ,009667 |
|  | 3 | 1 | ,0690000^*^ | ,0152096 | ,009 | ,022333 | ,115667 |
|  |  | 2 | ,0370000 | ,0152096 | ,111 | -,009667 | ,083667 |
| *. The mean difference is significant at the 0.05 level. | | | | | | | |

**Homogeneous Subsets**

| **Ace** | | | |
| --- | --- | --- | --- |
| Tukey HSD | | | |
| fact | N | Subset for alpha = 0.05 | |
|  |  | 1 | 2 |
| 3 | 3 | 14,0000 |  |
| 2 | 3 | 18,0000 |  |
| 1 | 3 |  | 26,0000 |
| Sig. |  | ,200 | 1,000 |
| Means for groups in homogeneous subsets are displayed. | | | |
| a. Uses Harmonic Mean Sample Size = 3,000. | | | |

| **CatH** | | | |
| --- | --- | --- | --- |
| Tukey HSD | | | |
| fact | N | Subset for alpha = 0.05 | |
|  |  | 1 | 2 |
| 1 | 3 | ,014000 |  |
| 2 | 3 | ,019000 | ,019000 |
| 3 | 3 |  | ,028000 |
| Sig. |  | ,420 | ,111 |
| Means for groups in homogeneous subsets are displayed. | | | |
| a. Uses Harmonic Mean Sample Size = 3,000. | | | |

| **CatG** | | |
| --- | --- | --- |
| Tukey HSD | | |
| fact | N | Subset for alpha = 0.05 |
|  |  | 1 |
| 1 | 3 | ,039000 |
| 2 | 3 | ,041000 |
| 3 | 3 | ,045000 |
| Sig. |  | ,280 |
| Means for groups in homogeneous subsets are displayed. | | |
| a. Uses Harmonic Mean Sample Size = 3,000. | | |

| **CatM** | | |
| --- | --- | --- |
| Tukey HSD | | |
| fact | N | Subset for alpha = 0.05 |
|  |  | 1 |
| 1 | 3 | ,011000 |
| 2 | 3 | ,012000 |
| 3 | 3 | ,014000 |
| Sig. |  | ,471 |
| Means for groups in homogeneous subsets are displayed. | | |
| a. Uses Harmonic Mean Sample Size = 3,000. | | |

| **GpxH** | | |
| --- | --- | --- |
| Tukey HSD | | |
| fact | N | Subset for alpha = 0.05 |
|  |  | 1 |
| 1 | 3 | 8,4000 |
| 2 | 3 | 10,2000 |
| 3 | 3 | 11,7000 |
| Sig. |  | ,137 |
| Means for groups in homogeneous subsets are displayed. | | |
| a. Uses Harmonic Mean Sample Size = 3,000. | | |

| **GpxG** | | |
| --- | --- | --- |
| Tukey HSD | | |
| fact | N | Subset for alpha = 0.05 |
|  |  | 1 |
| 1 | 3 | 9,8000 |
| 2 | 3 | 10,4000 |
| 3 | 3 | 11,6000 |
| Sig. |  | ,620 |
| Means for groups in homogeneous subsets are displayed. | | |
| a. Uses Harmonic Mean Sample Size = 3,000. | | |

| **GpxM** | | |
| --- | --- | --- |
| Tukey HSD | | |
| fact | N | Subset for alpha = 0.05 |
|  |  | 1 |
| 1 | 3 | 7,4000 |
| 2 | 3 | 8,1000 |
| 3 | 3 | 9,3000 |
| Sig. |  | ,309 |
| Means for groups in homogeneous subsets are displayed. | | |
| a. Uses Harmonic Mean Sample Size = 3,000. | | |

| **GSTH** | | |
| --- | --- | --- |
| Tukey HSD | | |
| fact | N | Subset for alpha = 0.05 |
|  |  | 1 |
| 1 | 3 | 37,2000 |
| 2 | 3 | 45,6000 |
| 3 | 3 | 52,3000 |
| Sig. |  | ,088 |
| Means for groups in homogeneous subsets are displayed. | | |
| a. Uses Harmonic Mean Sample Size = 3,000. | | |

| **GSTG** | | |
| --- | --- | --- |
| Tukey HSD | | |
| fact | N | Subset for alpha = 0.05 |
|  |  | 1 |
| 1 | 3 | 126,2000 |
| 2 | 3 | 132,2000 |
| 3 | 3 | 143,2000 |
| Sig. |  | ,205 |
| Means for groups in homogeneous subsets are displayed. | | |
| a. Uses Harmonic Mean Sample Size = 3,000. | | |

| **GSTM** | | |
| --- | --- | --- |
| Tukey HSD | | |
| fact | N | Subset for alpha = 0.05 |
|  |  | 1 |
| 1 | 3 | 33,4000 |
| 2 | 3 | 41,4000 |
| 3 | 3 | 43,2000 |
| Sig. |  | ,112 |
| Means for groups in homogeneous subsets are displayed. | | |
| a. Uses Harmonic Mean Sample Size = 3,000. | | |

| **LPOH** | | |
| --- | --- | --- |
| Tukey HSD | | |
| fact | N | Subset for alpha = 0.05 |
|  |  | 1 |
| 1 | 3 | 2,3000 |
| 2 | 3 | 3,5000 |
| 3 | 3 | 4,1000 |
| Sig. |  | ,070 |
| Means for groups in homogeneous subsets are displayed. | | |
| a. Uses Harmonic Mean Sample Size = 3,000. | | |

| **LPOG** | | | |
| --- | --- | --- | --- |
| Tukey HSD | | | |
| fact | N | Subset for alpha = 0.05 | |
|  |  | 1 | 2 |
| 1 | 3 | ,083000 |  |
| 2 | 3 | ,105000 | ,105000 |
| 3 | 3 |  | ,151000 |
| Sig. |  | ,511 | ,109 |
| Means for groups in homogeneous subsets are displayed. | | | |
| a. Uses Harmonic Mean Sample Size = 3,000. | | | |

| **LPOM** | | | |
| --- | --- | --- | --- |
| Tukey HSD | | | |
| fact | N | Subset for alpha = 0.05 | |
|  |  | 1 | 2 |
| 1 | 3 | ,062000 |  |
| 2 | 3 | ,094000 | ,094000 |
| 3 | 3 |  | ,131000 |
| Sig. |  | ,169 | ,111 |
| Means for groups in homogeneous subsets are displayed. | | | |
| a. Uses Harmonic Mean Sample Size = 3,000. | | | |
