# Supplementary material for: Effects of chlorpyrifos on the crustacean Litopenaeus vannamei
Source: PLoS One. 2020 Apr 13;15(4):e0231310. doi: 10.1371/journal.pone.0231310 (PMC7153863; doi:10.1371/journal.pone.0231310)
Supplement: Attachment — Submitted filename: answer to Plos one -Edisson 101019.docx [file pone.0231310.s005.docx]

Cartagena of Indias, 6 of November /2019

Staff

PLOS ONE

Kind regards.

 The authors appreciate the important comments from the academic Editor. Below,
We explain the reasons that justify the publication of this article entitled: Effects of Chlorpyrifos on the crustacean *Litopenaeus vannamei*.

.

 Thanks so much for your valuable collaboration.

Sincerely.

Beatriz E. Jaramillo C., Chem, Ph.D

Chemistry Program, Agrochemical Research Group, University of Cartagena, Cartagena of Indias, Colombia.

The Academic Editor Wrote:

The Academic Editor Wrote:

“The target chemical Chlorpyrifos is a traditional pesticide and its molecular mechanism of toxicity effect is relatively clear, and the study method is also traditional”.

Answer:

**We will write three reasons why the manuscript should be published**

1) Several research papers can be found on the effect of chlorpyrifos on mammals and fish, but much remains to be studied in aquatic species, especially in invertebrates (crustaceans). For example, when reviewing the topic with keywords such as chlorpyrifos and fish in www.pubmed.com, more than 400 articles appear, while searching in the same database with the keywords: “Litopenaeus vannamei” and “organophosphorus” and “chlorpyrifos” shows one result. We did a bibliographic tracking in the following databases: Sciencedirect, ACS, Scielo and Springer on Chlorpyrifos toxicity and we have found (see table 1)

Table 1. Articles found in the databases: Sciencedirect, ACS, Scielo and Springer on Chlorpyrifos toxicity

On invertebrates only exist 0,76% of public articles.

In the Stanford Encyclopedia of Philosophy we read:

“In developing ideas about the overall value of biodiversity it has been natural to draw on existing arguments about values of individual species (for review, see World Conservation Union 1980; Norton 1988). Commodity value and other direct use values have intuitive appeal because they reflect known values. But a key problem is that species need to be preserved for reasons other than any known value as resources for human use (Sober 1986). Callicott (1986) discusses philosophical arguments regarding non-utilitarian value and concludes that there is no easy argument to be made except a moral one. Species have some “intrinsic value” — reflecting the idea that a species has a value “in and for itself” (Callicott 1986, p.140) — and there is an ethical obligation to protect biodiversity”.

2) There are differences between the physiology of vertebrate and invertebrate organisms. In our study, the target organism was an invertebrate, crustacean, the Litopenaeus vannamei. See in the manuscript pages 3-4

Invertebrates do not have a spinal cord or spine; instead, most of them have an exoskeleton that spans the entire body. They do not have lungs since they breathe through their skin [1]. The liver participates mainly in the metabolism and regulation of nutrients in aquatic animals and plays an important role in oxidative stress and lipid peroxidation [2]. The crustaceans do not have liver; they have hepatopancreas, with functions similar to those of the fish liver (see fig1). Therefore, the investigation of possible biochemical changes in response to xenobiotics in crustaceans are done through the hepatopancreas [3], (see Fig. 1). However, the understanding of the influential mechanism underlying the pesticides in the hepatopancreas is still limited and needs further investigation [4]. So in Shrimp doesn’t exist a molecular mechanism of toxicity effect relatively clear.

Is also true that the vast majority of research is directed to biological models of vertebrate organisms, and less to invertebrates, such as *L. vannamei*, which precisely because of its economic importance and participation in the food of humans, must be ensured their food safety.

¿How chlorpyrifos affects a crustacean as the *Litopenaeus vannamei*?; what biomarkers could be used to assess its toxic effect

This research contributes to the study of the toxicity of chlorpyrifos in crustaceans, using the shrimp (*Litopenaeus vannamei*) as a model.

•	On the other hand, the cultivation of Shrimp, *Litopenaeus vannamei* has an important role in economic growth, employment and welfare of coastal communities, around the world. But is very sensible to xenobiotics like pesticides.

Fig 1. Anatomy of L. vannamei (<http://www.parasitosypatogenos.com.ar>).

1. Robalino J, Wilkins B, Bracken-Grissom HD, Chan T-Y, O’Leary MA The Origin of Large-Bodied Shrimp that Dominate Modern Global Aquaculture. PLoS One. 2016; 11(7): e0158840.
2. Lu Q, Sun Y, Ares I, Anadon A, Martinez M, Martínez-Larrañaga MR. Deltamethrin toxicity: A review of oxidative stress and metabolism. Environ. Res. 2019; 170: 260–281.
3. Silveira de Melo M, Gonçalves dos Santos TP, Jaramillo M, Nezzi L, Rauh Muller YM, Nazari EM. Histopathological and ultrastructural indices for the assessment of glyphosate-based herbicide cytotoxicity in decapod crustacean hepatopancreas. Aquat. toxicol. 2019; 210:207-214.
4. Yang Z, Zhang Y, Jiang Y, Zhu F, Zeng L, Wang Y, et al. Transcriptional responses in the hepatopancreas of Eriocheir sinensis exposed to deltamethrin. PLoS ONE. 2017; 12(9): e0184581.
3) Chlorpyrifos is one of the most utilized insecticides in Colombia for agricultural and domestic pest control, but this also results in non-target organisms exposed to either lethal or sublethal concentrations of this contaminant (García-de la Parra et al., 2006). In Colombia is permit the use the chlorpyrifos, here don’t exist none prohibition. We want to change Colombian lays about concentration limits chlorpyrifos. But politicians think that in Colombia, there is not enough research on this pesticide In Colombia, we need to disseminate research on the effects of chlorpyrifos to pressure government agencies and thus transform the laws that govern the use of this pesticide. With this work we would like to contribute to that objective. (see fig 2 and fig3)

Fig 2. Pesticide production in Colombia

Fig 3. Sales of Chlorpyrifos in Colombia

https://www.ica.gov.co/areas/agricola/servicios/regulacion-y-control-de-plaguicidas-quimicos/estadisticas/boletinplaguicidas2015-12-12-2016.aspx

This work is addressing a problem that could be considered at the regional level, but in reality, it is a global problem. The Bay of Cartagena and its biota has been exposed to a chlorpyrifos spill, and some companies (Dow Agrosciencies, Monsanto (Syngenta) produce this compound even in its industrial zone. See fig 4.

Fig 4. Chlorpyrifos spill in Cartagena (Colombia)

Fig 5. Plaguicides factories in Cartagena Bay (Colombia)

This factories to this day continue to pollute the marine environmental of Cartagena bay with Chlorpirifos (LORSBAN)

| Year | article |
| --- | --- |
| 1991 | Cowgill RT, Gowland CA, Ramirez V. The history of a chlorpyrifos spill:  Cartagena, Colombia. Environ. Int. 1991; 17: 61-71. |
| 2013 | https://sostenibilidad.semana.com/actualidad/articulo/cartagena-dano-ambiental-petroleo-dow-quimica/30230 |
| 2015 | <https://www.eltiempo.com/archivo/documento/CMS-15869078> |
| 2015 | https://www.javerianacali.edu.co/sites/ujc/files/node/field-documents/field_document_file/caso_presentado_por_estudiantes_derecho_javeriana_cali_en_buenos_aires.pdf |

Agrochemical Research Group, University of Cartagena, did a work where sampled marine sediments in nine sites in the Bay of Cartagena in collaboration with the University of Buffalo (New York) and the following concentrations of pesticides were found:

Fig 6. Pesticides concentration in Cartagena bay

The samples were conducted in June 2015 and 2017. We can see in the figure 6 that in seven of the sampling sites the highest concentration pesticide is chlorpiryfos.
