# Supplementary material for: Effects of chlorpyrifos on the crustacean Litopenaeus vannamei
Source: PLoS One. 2020 Apr 13;15(4):e0231310. doi: 10.1371/journal.pone.0231310 (PMC7153863; doi:10.1371/journal.pone.0231310)
Supplement: Attachment — Submitted filename: Response reviewers Plos One 16-02-20.docx [file pone.0231310.s006.docx]

Cartagena of Indias, 16 February 2020

Sir/Madam

Editor
**PLOS ONE**

Kind regards.

I´m sending you the answers to the reviewers, and corrections of the manuscript titled:” Effects of chlorpyrifos on the crustacean *Litopenaeus vannamei*”.

 The authors appreciate the important comments from the reviewers. Below, we explain the corrections and changes made to the article. The changes are indicated in the manuscript with red letters.

Thanks so much for your valuable collaboration.

Sincerely.

Beatriz Eugenia Jaramillo-Colorado., Chem, Ph.D

Chemistry Program, Agrochemical Research Group, University of Cartagena, Cartagena of Indias, Colombia.

**The Academic Editors Wrote:**

1. Please ensure that your manuscript meets PLOS ONE's style requirements, including those for file naming. The PLOS ONE style templates can be found at
   http://www.journals.plos.org/plosone/s/file?id=wjVg/PLOSOne_formatting_sample_main_body.pdf and <http://www.journals.plos.org/plosone/s/file?id=ba62/PLOSOne_formatting_sample_title_authors_affiliations.pdf>

**ANSWER**

It was checked and corrected.

2. We note that Figure(s) [Anatomy of L. vannamei (http://www.parasitosypatogenos.com.ar)] in your submission contain copyrighted images. (1) present written permission from the copyright holder to publish these figures specifically under the CC BY 4.0 license, or (2) remove the figures from your submission:

ANSWER

That was changed. We made our own illustration, using the GIMP Software v2.8.14.

See Figure 3.

3. Please amend your current ethics statement to confirm that your named ethics committee Institutional Care and Use Committee (IACUC) specifically approved this study.

ANSWER
The files will be loaded in the submit

4. Thank you for updating your data availability statement. You note that your data are available within the Supporting Information files, but no such files have been included with your submission. At this time we ask that you please upload your minimal data set as a Supporting Information file, or to a public repository such as Figshare or Dryad.
   Please also ensure that when you upload your file you include separate captions for your supplementary files at the end of your manuscript.

ANSWER

The Supporting information was added to the manuscript, and the files will be upload in the submission.

**REVIEWERS´ COMMENTS**

1. Have the authors made all data underlying the findings in their manuscript fully available?

ANSWER.

The Supporting information will be upload in the submission.

2. Is the manuscript presented in an intelligible fashion and written in standard English?

ANSWER

The manuscript was checked and corrected by an English language professional.

**Reviewer #1:**

3. Figures legends were all placed oddly throughout the manuscript and do a very poor job of describing the figures.

ANSWER

This was reviewed and corrected throughout the manuscript.

4. Table 1 has values that do not match the values in the manuscript.
   ANSWER

It was our mistake. Since we did not consider a unit conversión. This was corrected. See Table 1, and lines 235 to 242, page 10.

5. In the methods, it is not clear how many shrimp were placed in each replicate.

ANSWER

It was corrected. In toxicity tests, eight adult shrimps were used in each replicate. Please, to see page 5 line 124. In Sublethal toxicity assay Three replicates were done using five shrimps each. See page 6 line 151.

6. Reference #25 has the incorrect year.

ANSWER.

It was corrected. The year is 2015. See page 19, line 481

7. I could not find if the authors made all data underlying the findings in their manuscript fully available. That does not mean it is not there, I just do not know where to find it.

ANSWER.

The Supporting information was added to the manuscript, and the files will be upload in the submission. See page 15.

**Reviewer #2**
8. I think the manuscript is worth publishing whenever it is satisfactorily revised.
ANSWER

Thanks so much. Below, I describe the changes done.

8. Line 38: Last line in this paragraph seems inappropriately placed as no information on oxidative stress has been addressed at this point. Remove or re-word.
9. ANSWER.

It was removed.

10. Line 102: Was shrimp sex determined or was sex not considered during selection of organisms? This is unclear.

ANSWER

For this study, the gender of *L. vannamei* was not considerated. See page page 5 line 110.

10. Line 107: More detail on the brand or content of the shrimp feed may be useful.

ANSWER.

The feed was shrimp diet (Solla, Colombia). See Page 5, line 108

11. Line 108: There are many stages of intermoult in a shrimp's life. Clarify if the shrimp were at a certain life stage or state that they were not currently undergoing moult. "The intermoult stage" is unclear.

ANSWER

Shrimps were not currently undergoing moults. See Page 5, line 111.

11. Line 119: What type of plastic? Could this plastic affect your results at the temperature you held the shrimp at?

ANSWER.

The plastic was polypropylene. See page 5, line 106. It does not affect the experiment, since it was carried out at room temprature.

12. Line 218: Why were calibration curves so high (100-5000ug/L)? Test concentrations were only 0.7-13.0 ug/L. Clarify.

ANSWER

It was our mistake. Since we did not consider a unit conversión. This was corrected. See lines 219- 223, page 9.

13. Line 229: LC50 value in this line (2.10 ug/L) differs from the LC50 value in Table 1 (0.00021 ug/L). Confirm units.

ANSWER

It was our mistake. Since we did not consider a unit conversión. This was corrected. See Table 1, and lines 235 to 255, page 10.

14. Line 268: Behaviours are mentioned but observation of behaviour is not outlined in methods.

ANSWER.

It was removed.

15. Line 303: Was chlorpyrifos detected in muscle tissue? This is worded poorly and should be clarified. If stating a dose-dependent relationship then re-word the statement. If indeed the chlorpyrifos content of the muscle increased, include in methods how this was measured.

ANSWER

That was misspelled. Chlorpyrifos was not determined in the muscle. What was evaluated was the activity of catalase in the muscle. See page 12 line 305.
